# Effects of Single, Maximal Intensity Exercise Unit on Selected Markers of Bone and Connective Tissue Turnover in Young Men

**DOI:** 10.3390/jcm15124662

**Published:** 2026-06-16

**Authors:** Małgorzata Bagińska, Małgorzata Morawska-Tota, Tomasz Pałka, Łukasz Tota

**Affiliations:** 1 Department of Water Sports, Institute of Sport Sciences, University of Physical Culture in Kraków, 31-571 Kraków, Poland; 2 Department of Sports Medicine and Human Nutrition, Institute of Biomedical Sciences, University of Physical Culture in Kraków, 31-571 Kraków, Poland; 3 Department of Physiology and Biochemistry, Institute of Biomedical Sciences, University of Physical Culture in Kraków, 31-571 Kraków, Poland

**Keywords:** bone turnover markers, maximal exercise, bone metabolism, training status, young men

## Abstract

**Background/Objectives**: Bone tissue undergoes continuous remodelling processes that are closely controlled by mechanical stimuli and metabolic as well as inflammatory factors. Although the beneficial effects of regular physical activity on skeletal health are well documented, the response of bone turnover markers to a single maximal intensity exercise—particularly depending on training level—still remains ambiguous. The aim of the study was to assess the effect of a single physical exercise unit at maximum intensity on changes in selected biochemical markers specific for bone tissue, connective tissue remodelling and systemic stress response in young men. **Methods**: The study comprised 34 healthy men aged 20–25 years, divided into two groups: experimental (training, *n* = 15) and control (non-training, *n* = 19). The test was performed on a mechanical treadmill with a gradually increasing load (until subjective feeling of fatigue). Blood samples were collected before and 60 min following the test, and then assessed for the concentration of selected bone turnover markers, including bone-specific alkaline phosphatase (BSP), C-terminal telopeptide of type I collagen (CTX-I), deoxypyridinoline (DPD), hydroxyproline (GEN HYP) and haptoglobin (HPT). **Results**: In the group of training individuals, a significant decrease in BSP concentration was observed after physical exercise (*p* = 0.003). CTX-I concentration was significantly lower in training individuals compared to their non-training peers, regardless of measurement time (*p* < 0.001). Furthermore, a significant increase in GEN HYP concentration was observed after exercise in both groups (*p* = 0.033). For the remaining analysed markers, including DPD and HPT, no significant changes were demonstrated post-exercise. **Conclusions**: A single bout of maximal intensity exercise induces short-term, marker-specific changes in bone turnover dependent on training level, which reflect an early metabolic response of the musculoskeletal system rather than direct structural adaptation of the bone tissue.

## 1. Introduction

The human skeleton—as the body’s supporting structure—performs key metabolic, hematopoietic, motor and protective functions [1,2,3]. To effectively fulfil this role, bone tissue must be both strong and relatively light [4]. The structural integrity and strength of the tissue are maintained by the process of bone turnover—a dynamic and precisely regulated cellular mechanism that remains sensitive to both mechanical loads and hormonal changes while maintaining a state of dynamic balance [5]. The process of bone remodelling is regulated by many factors, such as age, sex, somatic indices (body mass and height, body composition, BMI), diet, taken medications and stimulants, the presence of diseases, genetic predispositions and level of physical activity [6,7,8].

Physical activity is widely recognised as an effective, non-pharmacological strategy for supporting skeletal health [9,10,11,12]. The anabolic effect of physical activity on bone tissue correlates positively with the intensity and nature of the interacting mechanical forces [13,14]. In previous studies, it has been shown that effective adaptation associated with bone remodelling requires dynamic and intermittent loading, whereas static stimuli do not lead to significant adaptive changes [7,15,16,17]. This means that not every type of training load will stimulate bone parameter growth and remodelling to an analogous extent [18].

Importantly, even very short periods of loading can initiate an adaptive response in the bone tissue due to the threshold nature of bone formation, which is modulated by factors such as the rate of deformation growth, its frequency, and amplitude and duration of the mechanical stimulus [18,19,20]. Mechanical loads generated during physical exercise cause deformation of the bone matrix, which leads to dynamic flow of interstitial fluid in the osteocytic lacuna–canaliculi system of the bones. The movement of this fluid generates shear forces acting on osteocyte protrusions, activating mechanotransduction and initiating a response that regulates bone turnover [21,22,23].

To fully utilise the potential of physical activity in skeletal health, it is essential to deepen our understanding regarding the metabolic responses of bone tissue to various types of mechanical stimuli. Comprehending the mechanisms regulating bone mineralisation can help prevent and reduce the risk of mechanical injuries [24,25]. Changes occurring in bone tissue depend on the nature and parameters of the mechanical load generated during physical activity. Regular, appropriately tailored exercise initiates local adaptations of bone tissue in areas exposed to mechanical forces, but the remodelling process varies depending on the type of stimulus used, its intensity, strength and duration [13,26]. Although sports can have a beneficial effect on bone health, long-term overload resulting from excessive training intensity and volume can lead to impaired bone metabolism, increasing the risk of repetitive strain injuries [27,28,29]. In turn, insufficient physical activity, a sedentary lifestyle and immobilisation are strongly associated with reduced bone mass and impaired bone mineralisation [30,31,32]. Although bone mineral density (BMD) remains a widely used indicator of bone health and a predictor of fracture risk [33], it only reflects the static level of mineralisation and does not allow for the identification of short-term metabolic changes induced by maximal physical activity. In this context, bone turnover markers (BTMs) are a valuable tool used to assess dynamic bone remodelling processes. Evaluating the concentration of bone turnover markers provides information on the activity of the two opposing processes of osteogenesis and osteolysis within bone tissue. Simultaneous determination of synthesis and resorption markers allows for the dynamics and intensity of these processes to be determined. This, in turn, may improve the identification of individuals at an increased risk of fracture [34,35].

Therefore, the aim of this study was to assess the effect of a single bout of maximal intensity exercise on changes in selected biochemical markers specific to bone tissue, connective tissue remodelling, and systemic stress response in young men undertaking varying levels of physical activity.

The following hypotheses were formulated:

Hypothesis 1: A single bout of maximal intensity exercise induces significant changes in markers of bone formation without a simultaneous increase in markers of bone resorption.

Hypothesis 2: Training status modulates the size and direction of acute, post-exercise biochemical responses, particularly for markers specific to bone tissue.

Hypothesis 3: A single bout of maximal intensity exercise differentiates the response of connective tissue remodelling markers as well as systemic stress response, especially in regularly training individuals. Non-bone-specific markers may reflect a broader musculoskeletal and systemic response.

## 2. Materials and Methods

This study is a supplement to/continuation of the author’s previous work on short-term changes in bone turnover markers in response to physical exercise—published in *PeerJ* [36]. This project has been extended to include additional biochemical markers.

### 2.1. Study Group

The study comprised 34 healthy men aged 20–25 years. The participants were classified into two groups based on their physical activity level. The training group (experimental group) (*n* = 15) consisted of individuals practicing middle- and long-distance running and achieving at least the II sports class. The control group (*n* = 19) did not engage in regular physical activity. These individuals could lead a sedentary lifestyle or refrain from regular physical activity for various reasons, provided their daily physical activity level did not exceed 3.0 METs, which corresponds to low-intensity exercise. To minimise the risk of bias, the non-training group was monitored throughout the entire protocol in identical conditions as that training. Detailed inclusion and exclusion criteria were presented in the author’s previous work [36].

The research project received a positive opinion from the Bioethics Committee at the Regional Medical Chamber in Kraków (No. 319/KBL/OIL/2021, date of approval: 5 November 2021). The study was conducted in accordance with the ethical standards of the Declaration of Helsinki. All participants signed written informed consent after reviewing the study protocol. In accordance with applicable standards, the research was conducted under the supervision of qualified medical personnel at the Central Scientific and Research Laboratory (CLNB) of the University of Physical Education in Kraków, PN-EN ISO 9001:2015 [37].

The project was funded by the Ministry of Science and Higher Education under the “Student Science Clubs Create Innovations” programme (No. SKN/SP/498248/2021). This prospective, controlled study was conducted from November 2021 during the 2021/2022 study period. The participants were enrolled in a three-day study protocol including repeated measurements before and after a single maximal intensity stress test.

### 2.2. Study Protocol

**Figure 1:**
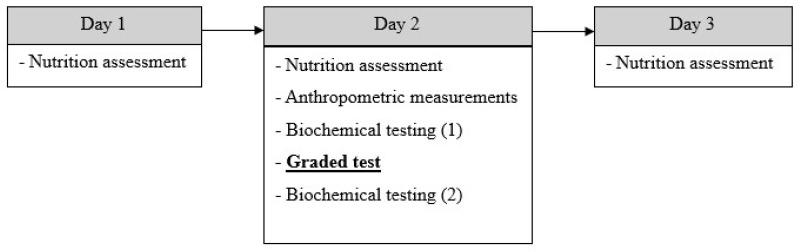
Scheme of study protocol. Legend: (1) First measurement; (2) Second measurement; arrows indicate the sequence of the study procedures.

The research procedure covered three measurement days and included:

- Nutrition assessment;
- Anthropometric measurements;
- Increasing-intensity stress test;
- Analysis of selected biochemical blood parameters (Figure 1).

### 2.3. Assessment of Nutrition Habits

Qualitative and quantitative assessments of the diet were conducted based on three-day food diaries covering the day before, the day of and that after its completion. Energy intake, macronutrient intake, and selected vitamins as well as minerals were calculated, comparing the values to current nutritional guidelines created by the National Institute of Public Health (PZH-PIB) [38]. The Aliant dietary calculator (EXPROMO, Wrocław, Poland, 2013–2026) was used for this purpose. During the study, the men did not change their eating habits or use any stimulants, vitamins or other supplements. Food diaries were verified and analysed under the supervision of qualified nutritionists.

### 2.4. Anthropometric Measurements

Before physiological and biochemical testing, selected somatic indices were evaluated. Body mass (BM) was measured using the certified SECA 875 medical scale (seca GmbH & Co. KG, Hamburg, Germany; accuracy 0.1 kg), and body height (BH) was estimated via the SECA 213 stadiometer (seca GmbH & Co. KG, Hamburg, Germany; accuracy 1 mm). Body composition was determined using the bioimpedance method (AKERN BIA 101, S.r.l., Pontassieve, Florence, Italy), including fat-free mass (FFM), fat mass (FM) and body fat percentage (FM%).

Anthropometric measurements were taken in the early morning hours (7:00–9:00 a.m.), before breakfast, in a state near euhydration and after passing morning urine.

Training and non-training participants were of similar body mass, with a slightly higher mean body height noted in the training group.

Differences between groups were primarily related to body composition, including fat-free body mass and body fat content, expressed in both absolute and percentage values [36].

### 2.5. Graded-Intensity Stress Test

Aerobic capacity was assessed directly using a test with an increasing workload on a mechanical treadmill (Saturn 250/100R, h/p/Cosmos Sports & Medical GmbH, Nussdorf-Traunstein, Germany) until refusal. Physiological indices obtained at the respiratory compensation point (RCP) and at the maximum level (VO_2_peak) were analysed.

Determination of the RCP was based on observations of changes in respiratory indices:

- Reaching the maximum, followed by a decrease in the fraction of CO_2_ in exhaled air;
- Reaching the minimum, followed by an increase in the respiratory equivalent value for CO_2_;
- Non-linear increase in lung ventilation after exceeding the RCP.

VO_2_peak was defined as the highest recorded value of oxygen uptake per minute [39].

The test began with a four-minute warm-up (8 km/h^−1^, 1° inclination), after which the speed was increased by 1 km/h^−1^ every two minutes until refusal due to exhaustion [40]. During the exercise, oxygen uptake, CO_2_ output, ventilation per minute, respiratory quotients and equivalents were recorded using the Cortex Metalyzer 3B ergospirometer from Germany. Heart rate (HR) was monitored using the Polar H7 transmitter from Finland.

Throughout the graded test and until the second blood collection (60 min after completing the test), the subjects did not consume any fluids or meals.

Detailed values of all analysed aerobic fitness indices were previously reported in a separate study conducted by the same authors [36].

### 2.6. Biochemical Blood Analysis

Venous blood was collected from the ulnar vein into EDTA tubes (Vacutainer) before and 60 min after the test was completed. The collection was carried out in certified laboratory conditions (PN-EN ISO 9001:2015 [37]). After centrifugation of the samples (2000 rpm, 15 min; MPW-351R, Poland), serum was obtained for analysis.

The concentrations of the analysed indices were determined via ELISA, using the Spark reader (Tecan, Switzerland) and ELK Biotechnology kits (Wuhan, China), considering their conceptual division into:

1. Bone-specific markers:
  ▪ Bone-specific alkaline phosphatase (BSP) (ELK1761);
  ▪ C-terminal telopeptide of type I collagen (CTX-I) (ELK2099);
  ▪ Deoxypyridinoline (DPD) (ELK9386).
2. Connective tissue remodelling markers:
  ▪ Hydroxyproline (GEN HYP) (ELK7835).
3. Systemic stress/inflammatory markers:
  ▪ Haptoglobin (HPT)(ELK1054).

Intra-assay precision (precision within an assay): CV% < 8%. Three samples of known concentration were tested twenty times on one plate to assess intra-assay precision. Inter-assay precision (precision between assays): CV% < 10%. Three samples of known concentration were tested in forty separate assays to assess inter-assay precision.

### 2.7. Statistical Analysis

Sample size was calculated using G*Power 3.1.9.7 (Germany). Statistical analysis was performed using IBM SPSS Statistics, version 28 (IBM Corp., Armonk, NY, USA). Normality of variable distribution and the residuals in the models were analysed using the Shapiro–Wilk test. The level of statistical significance in all analyses was set at α = 0.05.

Concerning the hypotheses on changes in concentrations of bone-specific alkaline phosphatase (BSP) levels—due to a violation of the normality assumption—we abandoned two-way analysis of variance in favour of using the Wilcoxon signed-rank test for dependent samples in each group separately, comparing the index values between the two measurement points. The effect size was evaluated using the r index, according to the convention: 0.10—weak effect; 0.30—moderate; >0.50—strong.

To verify changes in the concentration of C-terminal telopeptide of type I collagen (CTX-I), two-way analysis of variance was performed in a mixed model: 2 (group: athletes vs. non-training) × 2 (measurement time: before exercise vs. after exercise). The effect size was determined based on ηp^2^, with the following interpretation: 0.01—weak effect; 0.06—moderate; >0.14—strong. For the deoxypyridinoline (DPD) index—due to the lack of normality—the Wilcoxon signed-rank test was also applied for dependent samples in each group separately. The r index was implemented to assess the effect size, as interpreted above.

Analysis of changes in total hydroxyproline (GEN HYP) concentration was performed using a two-way mixed model analysis of variance: 2 (group) × 2 (time point), with effect size reported as ηp^2^. The normality of the residual distribution was verified using the Shapiro–Wilk test.

For changes in haptoglobin (HPT) concentration, the violation of normality resulted in the use of the Wilcoxon signed-rank test for dependent samples in each group separately to compare the two time points. The effect size was determined by the r index.

During the preparation of the cover letter, the authors used ChatGPT (OpenAI, GPT-5.5) for language editing and linguistic refinement. The authors have reviewed and edited the output and take full responsibility for the content of this publication.

## 3. Results

Among the training males, a decrease in BSP concentration was observed after exercise testing, with no significant changes in CTX-I, while GEN HYP concentrations tended to increase post-exercise and HPT concentrations slightly decreased. In the non-training group, mean BSP and CTX-I values remained stable between measurements, while DPD and GEN HYP demonstrated a minor increase after exercise. HPT concentrations did not change significantly. Detailed data are presented in Table 1.

In the group of training individuals, a significant decrease in BSP levels was observed between the first and second measurements (*p* = 0.003). Furthermore, a significant main effect of group was demonstrated for CTX-I (η^2^p = 0.867), with training individuals exhibiting significantly lower levels of this marker compared to non-training participants (F(1, 32) = 209.116, *p* < 0.001). For GEN HYP, a significant increase in the marker concentration was noted in the second measurement, regardless of group assignment (F(1, 32) = 4.937, *p* = 0.033; η^2^p = 0.134). For the remaining analysed bone turnover markers, no statistically significant effects were found (Table 2). There was also no effect of diet on changes in bone turnover markers.

## 4. Discussion

The aim of this study was to assess the effect of a single unit, maximal intensity physical exercise on changes in selected biochemical markers specific for bone tissue, connective tissue remodelling and systemic stress response in young men presenting different levels of physical activity. The results of the study partially confirmed the hypothesis, indicating that a single bout of maximal exercise does not induce a clear anabolic response in bone tissue, but leads to short-term changes in the profile of bone turnover markers, dependent on training level.

In response to the stress test, a significant 29.8% decrease in BSP concentration was observed in the group of individuals regularly engaging in physical activity (training), with no similar changes in the non-training group (0.6%). The decrease in bone-specific alkaline phosphatase concentration (*p* = 0.003) in our study among training individuals seems to contradict the classically accepted theory regarding the anabolic effect of mechanical loads on bone tissue [41]. However, the obtained result is consistent with the concept increasingly cited in the literature that acute responses of bone turnover markers do not necessarily directly reflect the direction of long-term structural adaptation [42,43,44]. Considering studies published in recent years, it has been pointed out that BSP and other bone formation markers may undergo a transient decrease immediately after vigorous exercise, which is explained, among others, by redistribution of blood flow, changes in plasma volume and increased uptake of markers by bone tissue and other organs in the early post-exercise response phase [8,39,42]. Such a transient effect may not be limited to bone markers. Davies et al. (2024) demonstrated that a single bout of moderate-intensity treadmill exercise transiently increased circulating 25(OH)D and 1,25(OH)2D3 concentrations compared to resting conditions, and this effect persisted for up to one hour post-exercise. These results highlight the importance of considering haemoconcentration and fluid shifts when interpreting post-exercise changes in any serum biomarker [45]. This may potentially lead to a transient decrease in their concentration in peripheral blood. Due to plasma volume changes not being assessed in the present study, it cannot be ruled out that some of the observed biomarker responses resulted from exercise-induced haemoconcentration or subsequent haemodilution. Maximal exercise intensity is known to induce rapid fluid shifts between the intra- and extravascular compartments, which may alter circulating biomarker concentrations, regardless of actual metabolic activity [45]. It is worth noting that the direction of the observed changes is not consistent with an effect resulting solely from haemoconcentration: the decrease in BSP concentration in the training group is opposite to the expected increase associated with fluid loss [46], which supports true metabolic inhibition of bone formation. In turn, the slight increase in HYP GEN concentration in both groups could have been partially exacerbated by haemoconcentration [47]. The above considerations emphasise the need for cautious interpretation of biomarker concentrations measured immediately after physical exercise.

The described mechanism is consistent with the concept of acute post-exercise changes in the distribution of bone turnover markers between systemic compartments, also observed for other bone turnover indices [39]. The decrease in BSP concentration noted only in the case of training individuals may reflect a different physiological response of athletes to maximal exercise, associated with faster and more intense cardiometabolic reactions [48]. Data found in the available data allow to suggest that in physically active individuals, acute changes in bone markers are more variable and more strongly dependent on the timing of sample collection than in inactive individuals, which limits the interpretation of individual BSP measurements in the immediate post-exercise period [49]. The significant main group effect for CTX-I (*p* < 0.001), with no effect of measurement timing or group × time interaction, indicates persistent differences in bone resorption between training and non-training individuals, independent of the acute exercise stimulus. The significantly lower CTX-I concentration in athletes compared to non-athletes (pre 0.899 ng/mL vs. 11.160 ng/mL; post 0.889 ng/mL vs. 10.917 ng/mL) suggests adaptation of the skeletal system to regular mechanical loads, leading to a relative inhibition of resorption processes. The exceptionally large size of this intergroup difference (η^2^p = 0.867) likely reflects not only the acute response to physical exercise but also significant differences in chronic, load-dependent osteogenic homeostasis. Long-term exposure to axial and impact loading in endurance-trained individuals leads to decreased resting bone resorption [47,48]. In the case of the non-training group, a chronic sedentary lifestyle is associated with significantly elevated basal CTX-I concentrations, consistent with a skeletal system subjected to insufficient mechanical loading, in which osteoclast activity is increased via the RANKL/RANK/OPG pathway [49,50]. The exceptionally large size of this difference between groups requires consideration of additional factors that may influence CTX-I concentrations. The CTX-I marker is particularly susceptible to preanalytical variability, including circadian rhythm, nutritional status, and sample collection as well as preparation procedures, as emphasised in the National Bone Health Alliance recommendations for standardisation of CTX-I assays [50]. Although all blood samples in this study were collected under standardised conditions (morning, fasting), the potential influence of these preanalytical factors should be considered when interpreting the size of the observed difference between groups. This persistent, interindividual difference highlights the significance of physical activity history as one of the main determinants of bone resorption markers in young adults. Additionally, the relatively small size of the study group could have increased the influence of extreme values on the obtained effect size (η^2^p). Furthermore, in the available literature on acute changes regarding bone turnover markers as a response to physical exercise, significant heterogeneity is indicated, depending on the type of exercise, the subjects’ training level and the blood sampling protocol [42,51]. Therefore, the exceptionally large effect size (η^2^p = 0.867) should be interpreted as the result of several coexisting factors, including true biological adaptation to long-term mechanical loads, high sensitivity of CTX-I assays to preanalytical factors [50], and methodological considerations related to the study design, including the relatively small sample size. This position is supported by population studies in which it is indicated that physically active individuals have higher bone mass and better mineralised bone tissue compared to their inactive peers [52,53,54]. The observed main effect is consistent with the results obtained in recent cross-sectional and interventional studies, demonstrating a more favourable profile of resorption markers among young, regularly training males compared to those leading a sedentary lifestyle [42,50,55,56]. Interestingly, from the perspective of training periodisation, the type of mechanical stimulus—not solely the total exercise volume—appears to be crucial for CTX-I modulation. In low-impact disciplines (swimming, cycling), a beneficial profile of resorption markers is not always observed, confirming the limited osteogenic effectiveness of loads devoid of a gravity component [51,57]. In this context, lower CTX-I concentrations in the studied athletes can be interpreted as a result of specific adaptation to disciplines characterised by axial and impact loads [58]. The lack of significant changes in DPD concentration among both study groups, despite the observed upward trend by 14% in the training group and 13% in the non-training group, may be due to the delayed kinetics of bone resorption markers, which often reach peak values only several or more hours after ending physical exercise. In current reports, it is emphasised that the dynamics regarding the response of bone turnover biochemical markers are strongly dependent on the timing of sample collection, and a single measurement immediately post-exercise may not reflect the full course of metabolic changes occurring in bone tissue [42,59,60]. Similarly, Hilkens et al. (2024) demonstrated that a single high-impact jump exercise did not modulate serum P1NP or CTX-I concentrations over a 24 h recovery period in healthy adults, reinforcing the concept that a single acute mechanical stimulus—even a high-impact one—may be insufficient in inducing measurable changes in systemic markers of bone turnover without repeated or prolonged loading [59]. It should be underscored that only two blood sampling points were used in this study (before exercise and 60 min after its completion), which makes it impossible to characterise delayed biomarker peaks of the implemented biomarkers. Many bone turnover markers, including CTX-I, DPD and collagen synthesis markers, may reach their maximum values only several or more hours after exercise [42,60,61]. Therefore, the presented results reflect only the early metabolic response post-exercise and do not provide a full kinetic profile of the bone tissue remodelling process. Due to the fact that blood sampling provides only a narrow time window, there is a risk that the peak kinetic response of bone turnover markers may not be detected in some studies [42].

Increasing attention is being paid to the endocrine role of bone tissue, particularly through the action of osteokines, such as uncarboxylated osteocalcin, which may mediate exercise-induced metabolic communication between bone, muscle and adipose tissue [60]. Although osteocalcin was not analysed in the current study, the perception of bone as an active endocrine organ suggests that in future research, both classical bone turnover markers (BTMs) should be considered, as well as osteokin profiles to more fully assess the metabolic response to acute maximal exercise intensity. In the research, it has been highlighted that significant changes in haptoglobin concentration are primarily observed after prolonged endurance exercise or longer mesocycles, which are accompanied by a marked activation of the inflammatory axis. Short-term maximal exercise, however, only provides a subthreshold stimulus, insufficient to trigger a response cascade [61,62]. The lack of significant changes in HPT concentration in our study suggests that the response of this protein to bone turnover may require stronger or longer-lasting stress than that used in the study protocol.

The significant increase in HYP GEN concentration (*p* = 0.033) observed in the second measurement by 5.5% and 3.8% (respectively) in the training and non-training subjects—and regardless of group assignment—may indicate a response to an intense stress stimulus. Hydroxyproline, as a marker of collagen degradation, also reflects processes occurring in skeletal muscle, tendons and connective tissue [63,64]. The increase in HYP GEN concentration after short-term maximal exercise is likely to result from increased collagen remodelling in the soft tissues, while collagen degradation is regulated, among others, by metalloproteinases and inflammatory processes accompanying microdamage induced by high-intensity exercise [65,66]. This phenomenon may be additionally amplified by post-exercise changes in plasma volume and perfusion, which influences serum marker concentrations [67]. Thus, the observed concentration changes should be interpreted with caution as circulating responses, potentially mediated by haemoconcentration or haemodilution. Hydroxyproline and haptoglobin were interpreted as complementary markers reflecting broader musculoskeletal and systemic responses, rather than as indicators specific to bone turnover alone. Therefore, the increase in hydroxyproline concentration should be interpreted as an element of the entire musculoskeletal system’s adaptive response to maximal exercise and not as a change specific only to bone tissue. Similar conclusions are drawn from experimental studies using collagen markers after high-intensity exercise, emphasising their limited bone specificity in the acute post-exercise period [68].

Assessing the degree of adherence to nutritional guidelines for selected macro- and micronutrients is the subject of numerous studies conducted in various populations due to their crucial importance for maintaining proper metabolic functions and body homeostasis [69]. In this study, the lack of dietary influence on changes in bone turnover markers may indicate that, in conditions of single-session, maximal-intensity exercises, the dominant factor modulating the bone response is the mechanical stimulus itself. In recent reports, it has been suggested that the influence of nutrition on bone turnover markers is observed in longitudinal studies or in populations exposed to chronically low energy availability, rather than in acute exercise protocols [70,71,72].

## 5. Conclusions and Limitations

Among young men, a single bout of maximal intensity physical exercise leads to selective, marker-specific changes in bone turnover, the nature and dynamics of which depend on training level. The significant decrease in BSP concentration observed in training individuals indicates that the acute response of bone formation markers does not necessarily reflect the direction of long-term bone tissue adaptation but may result from transient changes in marker concentrations during the immediate post-exercise period. At the same time, the persistently lower CTX-I concentrations in training individuals, regardless of the measurement time, suggests favourable adaptation of the skeletal system to regular mechanical loads (the effect of training length), leading to a relative inhibition of resorption processes. The lack of significant changes in DPD and HPT with a simultaneous increase in hydroxyproline concentration in both groups suggests that short-term exercise initiates a non-specific collagen response in the entire musculoskeletal system. These results reflect early post-exercise circulating responses and not comprehensive metabolic adaptation of bone tissue. It is important to bear in mind that the response of bone tissue to maximal physical exercise requires consideration of training level, the kinetics of individual markers and a multi-point assay scheme. Consequently, the presented results reflect only the early metabolic response of the body to a maximum effort and do not constitute a complete characterisation of the dynamics of bone tissue remodelling processes.

A single maximal intensity exercise appears to induce acute, marker-specific biochemical changes; however, these short-term responses should not be interpreted as direct evidence of anabolic bone adaptation. Properly integrating an individually planned training unit of this nature can support the maintenance of favourable osteogenic balance. In coaching practice—programming such units—the overall training load should be considered in order to minimise the risk of adverse overload effects while simultaneously leveraging the potential osteotropic effects of maximal-intensity exercise.

A limitation of the study was the lack of assessment regarding the long-term effects of bone turnover markers, which prevented a clear evaluation of whether the observed anabolic influence was permanent or merely transient with regard to the post-exercise period. The use of only pre-exercise and 60 min post-exercise measurements significantly restricts the full interpretation regarding the kinetics of the responses related to the tested biomarkers. Limiting blood sampling to two time points prevented full analysis of the kinetics of bone turnover marker responses to a single maximal exercise intensity and did not allow for the identification of potential delayed peaks in their concentrations. For markers such as CTX-I and DPD, peak values may not occur until several or even more hours after exercise. An additional methodological limitation was the lack of correction for plasma volume changes, which could have influenced acute, post-exercise biomarker concentrations, regardless of the actual metabolic changes (this issue has been deliberated in the “Section 4”). Furthermore, the sample size was relatively small and should be considered minimal, limiting the generalisability of the results. Therefore, the obtained results should be interpreted with caution, and their confirmation requires further research addressing the described limitations.

## Figures and Tables

**Table 1:** Changes in concentrations of biochemical markers in response to physical exercise in studied groups.

Group		Marker	Measurement Before		Measurement After	
			M (SD)	95% CI	M (SD)	95% CI
Training (*n* = 15)	Bone-specific	BSP (pg/mL)	266.049 (93.830)	[214.088, 318.011]	186.821 (88.141)	[138.010, 235.631]
		CTX-I (ng/mL)	0.899 (0.378)	[0.691, 1.109]	0.889 (0.389)	[0.674, 1.105]
		DPD (ng/mL)	0.577 (0.310)	[0.405, 0.749]	0.658 (0.385)	[0.445, 0.871]
	Connective tissue remodelling	GEN HYP (ng/mL)	3674.42 (869.787)	[3192.74, 4156.09]	3876.09 (681.860)	[3498.49, 4253.69]
	Systemic stress/ inflammatory	HPT (ng/mL)	21.855 (15.891)	[13.055, 30.656]	20.071 (11.386)	[13.765, 26.376]
Non-training (*n* = 19)	Bone-specific	BSP (pg/mL)	217.626 (270.730)	[82.995, 352.256]	216.236 (292.812)	[70.624, 361.847]
		CTX-I (ng/mL)	11.160 (2.964)	[9.731, 12.589]	10.917 (2.603)	[9.662, 12.171]
		DPD (ng/mL)	1.835 (5.195)	[−0.670, 4.339]	2.074 (6.507)	[−1.062, 5.211]
	Connective tissue remodelling	GEN HYP (ng/mL)	3435.55 (743.354)	[3077.27, 3793.84]	3564.66 (617.682)	[3266.95, 3862.38]
	Systemic stress/ inflammatory	HPT (ng/mL)	24.585 (13.289)	[18.180, 30.991]	24.194 (9.253)	[19.734, 28.654]

BSP—bone-specific alkaline phosphatase; CTX-I—C-terminal telopeptide of type I collagen; DPD—deoxypyridinoline; GEN HYP—hydroxyproline; HPT—haptoglobin; M (SD)—arithmetic mean (standard deviation); 95% CI—95-percent confidence interval for mean.

**Table 2:** Results of statistical analyses for selected bone turnover markers.

	Marker	Analysis	Effect/Comparison	Statistic (df)	*p*-Value	Effect Size
Bone-specific	BSP	Wilcoxon signed-rank	Pre vs. Post (training)	Z = −2.953	0.003	r = 0.762
			Pre vs. Post (non-training)	Z = −0.327	0.744	r = 0.077
	CTX-I	ANOVA	Grupa	F(1, 32) = 209.116	<0.001	η^2^p = 0.867
			Moment of Measurement	F(1, 32) = 0.426	0.519	η^2^p = 0.013
			Grupa × Moment of measurement	F(1, 32) = 0.359	0.553	η^2^p = 0.011
	DPD	Wilcoxon signed-rank	Pre vs. Post (training)	Z = −1.193	0.233	r = 0.308
			Pre vs. Post (non-training)	Z = −0.282	0.778	r = 0.065
Connective tissue remodelling	GEN HYP	ANOVA	Group	F(1, 32) = 1.313	0.260	η^2^p = 0.039
			Moment of Measurement	F(1, 32) = 4.937	0.033	η^2^p = 0.134
			Grupa × Moment of measurement	F(1, 32) = 0.238	0.629	η^2^p = 0.007
Systemic stress/ inflammatory	HPT	Wilcoxon signed-rank	Pre vs. Post (training)	Z = −0.852	0.394	r = 0.220
			Pre vs. Post (non-training)	Z = −0.644	0.520	r = 0.148

BSP—bone-specific alkaline phosphatase; CTX-I—C-terminal telopeptide of type I collagen; DPD—deoxypyridinoline; GEN HYP—hydroxyproline; HPT—haptoglobin.

## Data Availability

Data are included in the article or the ‘Supplementary Materials’ section.

## Supplementary Materials

The following supporting information can be downloaded at: https://www.mdpi.com/article/10.3390/jcm15124662/s1, Table S1. Individual values of biochemical markers measured before and after exercise testing in training and non-training participants, including BSP, CTX-I, DPD, GEN HYP, and HPT concentrations; Table S2. Individual physiological responses obtained during the VO_2_max test, including maximal exercise values and respiratory compensation point (RCP) parameters in training and non-training participants; Table S3. Individual body composition characteristics of study participants, including body height (BH), body mass (BM), body cell mass (BCM), fat-free mass (FFM), fat mass (FM), total body water (TBW), and extracellular water (ECW).
