# Supplementary material for: Effects of Single, Maximal Intensity Exercise Unit on Selected Markers of Bone and Connective Tissue Turnover in Young Men
Source: J Clin Med. 2026 Jun 16;15(12):4662. doi: 10.3390/jcm15124662 (PMC13300812; doi:10.3390/jcm15124662)
Supplement: Supplementary file 1 [file jcm-15-04662-s001.zip › Supplementary Table S1. Individual values of biochemical markers measured before and after exercise testing in training and non-training participants, including BSP,.pdf]

	BSP (Bone-Specific Alkaline Phosphatase)			CTX1 (C-terminal Telopeptide of			DPD (Deoxypyridinolin)			GEN HYP (Hydroxyproline)			HPT (Haptoglobina)		
Training participants ID	BEFORE	AFTER	UNIT	BEFORE	AFTER	UNIT	BEFORE	AFTER	UNIT	BEFORE	AFTER	UNIT	BEFORE	AFTER	UNIT
1T	365,1279	211,9419	pg/ml	0,190424	0,246269	ng/mL	0,783259	0,700189	ng/ml	5084,94	4864,166	ng/ml	33,40982	53,48178	ng/ml
2T	327,1257	39,722662		0,623027	0,723939		0,390695	0,672376		4376,087	4134,586		10,80644	10,50988	
3T	276,5394	156,80727		1,241819	1,018588		0,363779	0,939458		2944,129	3357,593		17,24298	17,35289	
4T	182,5309	128,18248		0,616632	0,594266		0,283777	0,548235		4308,696	4704,92		19,43104	18,62978	
5T	135,5989	157,20823		0,891611	0,91677		0,30656	0,414105		2876,965	3196,246		15,33234	9,645779	
6T	242,8167	199,69644		0,725439	0,748104		0,817384	0,991359		2928,185	3089,899		17,56646	10,81008	
7T	231,0907	114,11414		0,244654	0,229579		0,565526	0,698953		5368,931	5071,298		31,67676	26,99409	
8T	341,9732	190,7355		1,071895	0,649799		0,595229	0,661967		4229,255	4398,225		10,51108	9,917063	
9T	477,7986	411,23533		0,993063	1,035695		0,76884	0,165548		3722,265	3800,606		74,06887	30,12622	
10T	335,1613	235,99028		1,254726	1,563088		0,55232	0,311837		3092,834	3037,001		18,24311	23,56706	
11T	291,7877	308,78447		1,400003	1,3583		0,528058	0,193743		3606,504	3784,535		21,15765	19,18475	
12T	219,9575	135,31057		0,796498	0,834327		0,361442	0,477513		3247,11	3942,868		13,49906	17,08727	
13T	253,7665	190,00236		0,857157	0,81191		1,505899	1,67094		4047,402	4292,546		17,27146	16,66538	
14T	174,0579	207,05226		1,193944	1,387568		0,364998	0,425906		2618,748	3291,3		12,02567	26,05845	
15T	135,4067	115,52488		1,396552	1,222358		0,465559	1,001624		2664,19	3175,597		15,58794	11,02661	

Non-training participants ID	BEFORE	AFTER	UNIT	BEFORE	AFTER	UNIT	BEFORE	AFTER	UNIT	BEFORE	AFTER	UNIT	BEFORE	AFTER	UNIT
1NT	126,1557	143,75447	pg/ml	10,62097	13,50323	ng/mL	0,72402	0,769688	ng/ml	3844,078	3842,534	ng/ml	12,11889	7,141434	ng/ml
2NT	239,4505	154,94348		10,08845	10,60293		0,303658	0,575896		4458,088	4632,47		22,27092	28,97415	
3NT	359,854	156,69785		7,510454	7,673708		0,48599	0,274982		4185,765	3134,347		24,94762	26,56975	
4NT	114,9328	168,34737		5,246845	5,304471		0,522098	0,51094		4152,119	4035,883		23,28489	35,38607	
5NT	105,2724	251,35085		12,33018	12,16924		0,601596	0,67705		2403,034	2583,917		20,18954	23,78165	
6NT	130,6848	124,05708		11,84323	13,77398		1,046302	0,906265		3091,452	3566,399		18,1688	21,06137	
7NT	59,77835	119,92007		13,91613	13,58027		0,41811	0,221929		3178,92	3625,716		16,36651	22,32647	
8NT	49,02247	105,46251		14,7545	12,86686		0,438768	0,015631		4413,661	4883,326		19,36378	17,97928	
9NT	150,748	190,7355		11,73135	12,82043		0,557276	0,153101		3201,507	3086,452		14,13606	12,92325	
10NT	291,7279	210,73943		8,044462	8,679767		0,292435	0,829389		3025,758	3163,96		35,17509	24,76888	
11NT	155,2913	244,62823		13,56744	13,07057		1,253663	1,175895		2939,229	2859,381		24,08226	18,79759	
12NT	295,5059	86,012364		10,36146	9,712844		0,585473	0,777419		3535,857	2965,8		26,27892	25,06449	
13NT	41,05398	0		9,146111	8,427897		1,56247	0,700189		4788,325	4216,41		16,64254	20,24769	
14NT	79,75206	45,803368		15,18267	11,60756		0,038021	0,662922		2625,22	3433,59		22,9094	23,74584	
15NT	78,69003	109,8831		13,20874	11,16346		0,947256	0,91613		2499,126	3214,441		32,01676	15,54702	
16NT	31,22812	43,214521		13,99866	13,81397		0,526691	0,736335		3512,24	3354,578		12,25588	20,5093	
17NT	118,8523	193,08202		14,03283	11,72478		0,844189	0,18312		2505,694	3430,474		18,88513	43,74902	
18NT	301,1711	178,15475		5,811522	6,224913		0,470107	0,412703		4064,515	4351,542		36,71065	43,99897	
19NT	1229,141	1365,4552		10,64672	10,69572		23,23716	28,91367		2850,916	3347,395		71,31837	27,1142	
