# Supplementary material for: Effects of Single, Maximal Intensity Exercise Unit on Selected Markers of Bone and Connective Tissue Turnover in Young Men
Source: J Clin Med. 2026 Jun 16;15(12):4662. doi: 10.3390/jcm15124662 (PMC13300812; doi:10.3390/jcm15124662)
Supplement: Supplementary file 1 [file jcm-15-04662-s001.zip › Supplementary Table S2. Individual physiological responses obtained during the VO2max test, including maximal exercise (MAX) and respiratory compensation point (RCP).pdf]

Training participants ID	MAX t [min]	v [km/h]	HR [bpm]	VO_2 [L·min-1]	VO_2/kg	Ve [l/min]	RCP t [min]	v [km/h]	HR [bpm]	VO_2 [L·min-1]	VO_2/kg	Ve [l/min]
1T	19,30	17,50	176,00	3,89	52,93	158,80	12,00	12,90	155,00	3,10	42,18	97,30
2T	16,30	15,30	181,00	3,75	50,81	162,50	10,00	11,80	166,00	3,32	44,99	121,40
3T	16,30	15,20	185,00	3,63	50,21	139,30	11,00	12,20	168,00	3,24	44,81	94,00
4T	19,00	16,70	177,00	4,36	56,70	194,40	10,00	11,80	160,00	3,53	45,90	120,00
5T	19,00	17,70	185,00	3,83	55,19	177,10	11,30	12,80	169,00	3,24	46,69	88,50
6T	11,00	10,30	174,00	4,13	43,94	142,90	9,30	10,80	161,00	3,87	41,17	112,90
7T	18,00	16,20	187,00	3,90	49,62	159,40	14,30	14,20	175,00	3,55	45,17	121,70
8T	22,30	19,20	189,00	4,47	60,73	184,90	14,30	14,80	172,00	3,95	53,67	122,10
9T	19,00	16,70	203,00	3,69	56,77	127,60	10,00	11,80	172,00	2,71	41,69	73,30
10T	16,30	16,40	183,00	3,84	46,94	155,80	10,30	12,70	169,00	3,40	41,56	108,70
11T	16,00	15,10	179,00	3,84	48,12	149,90	8,00	10,70	162,00	3,09	38,72	96,30
12T	16,30	15,30	190,00	3,88	47,49	140,00	10,00	11,90	156,00	3,16	38,68	78,00
13T	22,30	19,20	183,00	3,76	59,12	157,00	11,30	11,10	169,00	3,36	52,83	115,80
14T	16,30	14,00	196,00	3,88	49,94	143,30	10,00	9,00	173,00	2,85	36,68	89,90
15T	13,30	13,70	188,00	2,87	37,03	125,60	10,00	11,70	179,00	2,60	33,55	78,80
**Non-training participants ID**												
1NT	13,30	13,70	198,00	2,87	42,77	125,60	12,00	11,20	179,00	2,60	38,75	78,80
2NT	19,16	15,70	178,00	5,12	65,56	158,20	12,30	11,50	156,00	4,04	51,73	96,00
3NT	15,30	14,80	179,00	3,17	33,06	148,30	14,10	14,30	171,00	2,52	26,28	82,70
4NT	19,68	17,50	172,00	4,29	58,29	166,00	11,30	11,50	149,00	3,80	51,63	99,20
5NT	18,00	16,10	184,00	3,91	55,38	172,30	11,30	12,60	162,00	3,07	43,48	102,30
6NT	18,00	16,10	188,00	4,41	59,59	177,30	13,00	12,30	178,00	3,44	46,49	102,50
7NT	19,30	16,40	200,00	3,85	55,72	145,90	11,00	12,30	177,00	3,02	43,70	92,40
8NT	21,00	17,70	191,00	4,40	52,51	169,00	13,30	12,60	160,00	3,14	37,47	77,50
9NT	15,30	14,80	199,00	3,41	47,96	137,70	9,00	11,20	178,00	2,80	39,38	91,60
10NT	16,30	14,30	179,00	3,72	50,96	116,90	9,30	10,60	153,00	3,10	42,47	68,00
11NT	18,30	15,50	193,00	3,81	52,19	149,00	10,30	11,00	163,00	2,91	39,86	81,80
12NT	12,00	11,90	182,00	3,47	44,49	100,60	7,00	9,10	171,00	3,00	38,46	77,90
13NT	14,30	13,20	191,00	3,28	42,05	143,40	9,30	10,50	171,00	2,81	36,03	97,50
14NT	18,00	15,20	220,00	4,34	56,73	154,70	9,00	10,20	177,00	3,06	40,00	75,50
15NT	12,30	12,10	153,00	5,30	66,25	176,00		10,70	179,00	5,06	63,25	100,20
16NT	18,30	15,40	180,00	3,84	39,59	144,90	8,00	9,70	156,00	2,77	28,56	77,40
17NT	16,30	14,30	192,00	4,13	55,36	137,90	9,30	10,60	169,00	3,37	45,17	87,10
18NT	15,30	14,80	192,00	4,47	60,00	187,20	9,00	11,10	175,00	3,74	50,20	116,30
19NT	19,00	16,00	195,00	4,52	66,28	157,30	13,00	13,50	180,00	3,80	55,72	93,40
