# Supplementary material for: Effects of Single, Maximal Intensity Exercise Unit on Selected Markers of Bone and Connective Tissue Turnover in Young Men
Source: J Clin Med. 2026 Jun 16;15(12):4662. doi: 10.3390/jcm15124662 (PMC13300812; doi:10.3390/jcm15124662)
Supplement: Supplementary file 1 [file jcm-15-04662-s001.zip › Supplementary Table S3. Individual body composition characteristics measured before and after the study period, including BH, BM, BCM, FFM, FM, TBW, and ECW..pdf]

| | BH [cm] | | BM [kg] | | BCM [kg] | | FFM [kg] | | FM [%] | | FM [kg] | | TBW [L] | | TBW [%] | | ECW [L] | |
|---|---|---|---|---|---|---|---|---|---|---|---|---|---|---|---|---|---|---|
| **Training participants ID** | **BEFORE** | **AFTER** | **BEFORE** | **AFTER** | **BEFORE** | **AFTER** | **BEFORE** | **AFTER** | **BEFORE** | **AFTER** | **BEFORE** | **AFTER** | **BEFORE** | **AFTER** | **BEFORE** | **AFTER** | **BEFORE** | **AFTER** |
| 1T | 179,5 | 179,5 | 73,5 | 73,8 | 34,0 | 33,1 | 59,8 | 58,7 | 18,6 | 20,5 | 13,7 | 15,1 | 43,7 | 42,9 | 59,5 | 58,1 | 18,7 | 18,5 |
| 2T | 186,5 | 186,5 | 73,8 | 71,9 | 30,9 | 31,5 | 58,3 | 58,9 | 21,0 | 18,1 | 15,5 | 13,0 | 42,7 | 43,2 | 57,9 | 60,1 | 19,8 | 19,9 |
| 3T | 186,4 | 186,4 | 72,3 | 73,9 | 34,5 | 34,5 | 59,8 | 61,5 | 17,3 | 16,8 | 12,5 | 12,4 | 43,6 | 45,0 | 60,3 | 60,9 | 18,4 | 19,6 |
| 4T | 186,2 | 186,2 | 76,9 | 76,8 | 34,1 | 36,7 | 63,9 | 68,2 | 16,9 | 11,2 | 13,0 | 8,6 | 46,9 | 50,3 | 61,0 | 65,5 | 21,6 | 22,9 |
| 5T | 181,6 | 181,6 | 69,4 | 69,4 | 34,2 | 34,2 | 58,3 | 58,3 | 16,0 | 16,0 | 11,1 | 11,1 | 42,5 | 42,5 | 61,2 | 61,2 | 17,5 | 17,5 |
| 6T | 184,0 | 184,0 | 94,0 | 95,6 | 38,7 | 37,5 | 68,9 | 70,1 | 26,7 | 26,7 | 25,1 | 25,5 | 50,5 | 51,6 | 53,7 | 54,0 | 22,0 | 23,7 |
| 7T | 180,0 | 180,0 | 78,6 | 78,6 | 37,4 | 37,4 | 63,9 | 63,9 | 18,7 | 18,7 | 14,7 | 14,7 | 46,7 | 46,7 | 59,4 | 59,4 | 19,3 | 19,3 |
| 8T | 183,0 | 183,0 | 73,6 | 72,3 | 37,7 | 38,1 | 64,4 | 64,3 | 12,5 | 11,1 | 9,2 | 8,0 | 47,2 | 47,1 | 64,1 | 65,1 | 19,5 | 19,2 |
| 9T | 176,0 | 176,0 | 65,0 | 65,0 | 33,5 | 34,7 | 58,3 | 60,0 | 10,3 | 7,7 | 6,7 | 5,0 | 42,7 | 44,0 | 65,7 | 67,7 | 18,1 | 18,5 |
| 10T | 183,5 | 183,5 | 81,8 | 81,8 | 37,6 | 37,6 | 67,0 | 67,0 | 18,1 | 18,1 | 14,8 | 14,8 | 49,2 | 49,2 | 60,1 | 60,1 | 21,4 | 21,4 |
| 11T | 178,0 | 178,0 | 79,8 | 80,9 | 39,5 | 39,3 | 64,0 | 65,8 | 19,8 | 18,7 | 15,8 | 15,1 | 46,6 | 48,1 | 58,4 | 59,5 | 18,0 | 19,4 |
| 12T | 183,0 | 183,0 | 81,7 | 80,8 | 39,3 | 39,3 | 68,8 | 67,0 | 15,8 | 17,1 | 12,9 | 13,8 | 50,5 | 49,1 | 61,8 | 60,8 | 21,6 | 20,3 |
| 13T | 176,5 | 176,5 | 63,6 | 64,8 | 33,5 | 34,4 | 57,0 | 59,8 | 10,4 | 7,7 | 6,6 | 5,0 | 41,6 | 43,8 | 65,4 | 67,6 | 17,1 | 18,5 |
| 14T | 184,0 | 184,0 | 77,7 | 77,0 | 40,1 | 40,4 | 64,9 | 66,4 | 16,5 | 13,8 | 12,8 | 10,6 | 47,3 | 48,5 | 60,9 | 63,0 | 18,2 | 19,0 |
| 15T | 179,0 | 179,0 | 77,5 | 79,1 | 36,8 | 38,5 | 61,4 | 65,6 | 20,8 | 17,1 | 16,1 | 13,5 | 44,8 | 48,1 | 57,8 | 60,8 | 18,0 | 19,8 |
| | | | | | | | | | | | | | | | | | | |
| **Non-training participants ID** | **BEFORE** | **AFTER** | **BEFORE** | **AFTER** | **BEFORE** | **AFTER** | **BEFORE** | **AFTER** | **BEFORE** | **AFTER** | **BEFORE** | **AFTER** | **BEFORE** | **AFTER** | **BEFORE** | **AFTER** | **BEFORE** | **AFTER** |
| 1NT | 169,5 | 169,5 | 67,1 | 66,3 | 33,8 | 35,3 | 56,5 | 57,8 | 15,8 | 12,8 | 10,6 | 8,5 | 41,2 | 42,1 | 61,4 | 63,5 | 16,6 | 16,5 |
| 2NT | 187,5 | 187,5 | 78,1 | 77,1 | 38,9 | 39,4 | 68,2 | 69,6 | 12,7 | 9,7 | 9,9 | 7,5 | 50,1 | 51,2 | 64,1 | 66,4 | 21,4 | 22,0 |
| 3NT | 187,0 | 187,0 | 95,9 | 97,2 | 49,0 | 50,7 | 83,4 | 84,6 | 13,0 | 13,0 | 12,5 | 12,6 | 61,5 | 62,4 | 64,1 | 64,2 | 25,3 | 25,1 |
| 4NT | 172,5 | 172,5 | 73,6 | 72,5 | 35,8 | 37,9 | 60,4 | 62,2 | 17,9 | 14,2 | 13,2 | 10,3 | 44,1 | 45,4 | 59,9 | 62,6 | 18,0 | 17,8 |
| 5NT | 175,5 | 175,5 | 70,6 | 70,4 | 37,9 | 41,3 | 63,4 | 65,3 | 10,2 | 7,2 | 7,2 | 5,1 | 46,4 | 47,7 | 65,7 | 67,8 | 18,7 | 17,8 |
| 6NT | 179,5 | 179,5 | 74,0 | 73,1 | 36,6 | 36,0 | 62,5 | 61,8 | 15,5 | 15,5 | 11,5 | 11,3 | 45,7 | 45,2 | 61,8 | 61,8 | 18,9 | 18,8 |
| 7NT | 171,5 | 171,5 | 69,1 | 69,2 | 35,2 | 36,1 | 59,4 | 59,5 | 14,0 | 14,0 | 9,7 | 9,7 | 43,4 | 43,4 | 62,8 | 62,7 | 17,7 | 17,1 |
| 8NT | 176,0 | 176,0 | 83,8 | 85,3 | 46,6 | 46,2 | 72,6 | 76,1 | 13,4 | 10,8 | 11,2 | 9,2 | 53,1 | 56,0 | 63,4 | 65,7 | 19,4 | 22,0 |
| 9NT | 173,0 | 173,0 | 71,1 | 71,1 | 39,8 | 39,8 | 65,6 | 65,6 | 7,7 | 7,7 | 5,5 | 5,5 | 48,1 | 48,1 | 67,7 | 67,7 | 19,0 | 19,0 |
| 10NT | 178,0 | 178,0 | 73,0 | 74,0 | 38,8 | 36,7 | 65,3 | 64,9 | 10,5 | 12,3 | 7,7 | 9,1 | 47,8 | 47,7 | 65,5 | 64,5 | 19,4 | 20,6 |
| 11NT | 178,0 | 178,0 | 73,0 | 72,6 | 38,8 | 40,7 | 65,3 | 67,9 | 10,5 | 6,5 | 7,7 | 4,7 | 47,8 | 49,8 | 65,5 | 68,6 | 19,4 | 20,0 |
| 12NT | 184,5 | 184,5 | 78,0 | 78,4 | 39,7 | 41,9 | 66,2 | 69,7 | 15,1 | 11,1 | 11,8 | 8,7 | 48,4 | 51,1 | 62,1 | 65,2 | 19,4 | 20,5 |
| 13NT | 187,0 | 187,0 | 78,0 | 78,0 | 40,4 | 38,9 | 66,2 | 65,6 | 15,1 | 15,9 | 11,8 | 12,4 | 48,3 | 48,0 | 62,0 | 61,5 | 19,0 | 19,6 |
| 14NT | 177,0 | 177,0 | 76,5 | 76,5 | 35,8 | 35,8 | 64,0 | 64,0 | 16,3 | 16,3 | 12,5 | 12,5 | 47,0 | 47,0 | 61,5 | 61,4 | 20,5 | 20,5 |
| 15NT | 173,0 | 173,0 | 80,0 | 78,4 | 39,1 | 39,7 | 68,6 | 66,8 | 14,3 | 14,8 | 11,4 | 11,6 | 50,5 | 49,0 | 63,1 | 62,5 | 21,6 | 19,8 |
| 16NT | 190,0 | 190,0 | 97,0 | 95,0 | 47,5 | 47,7 | 81,8 | 80,1 | 15,7 | 15,7 | 15,2 | 14,9 | 60,4 | 59,0 | 62,2 | 62,1 | 25,3 | 23,8 |
| 17NT | 191,5 | 191,5 | 74,6 | 74,9 | 38,8 | 40,0 | 69,1 | 69,9 | 7,4 | 6,7 | 5,5 | 5,0 | 50,8 | 51,4 | 68,1 | 68,6 | 22,1 | 21,9 |
| 18NT | 178,5 | 178,5 | 74,5 | 77,5 | 44,9 | 46,5 | 71,7 | 72,6 | 3,8 | 6,3 | 2,8 | 4,9 | 52,6 | 53,1 | 70,7 | 68,5 | 19,9 | 19,4 |
| 19NT | 172,0 | 172,0 | 68,2 | 67,6 | 38,1 | 37,8 | 64,1 | 61,7 | 6,0 | 8,7 | 4,1 | 5,9 | 47,1 | 45,1 | 69,1 | 66,7 | 19,1 | 17,5 |
